# Hydrous Hydrazine Decomposition for Hydrogen Production Using of Ir/CeO_2_: Effect of Reaction Parameters on the Activity

**DOI:** 10.3390/nano11051340

**Published:** 2021-05-19

**Authors:** Davide Motta, Ilaria Barlocco, Silvio Bellomi, Alberto Villa, Nikolaos Dimitratos

**Affiliations:** 1 Cardiff Catalysis Institute, School of Chemistry, Cardiff University, Cardiff CF10 3AT, UK; davide.motta90@gmail.com; 2 Dipartimento di Chimica, Università degli Studi di Milano, 20133 Milano, Italy; ilaria.barlocco@unimi.it (I.B.); silvio.bellomi@studenti.unimi.it (S.B.); 3 Dipartimento di Chimica Industriale e dei Materiali, Alma Mater Studiorum Università di Bologna, 40136 Bologna, Italy

**Keywords:** iridium, cerium oxide, hydrous hydrazine, hydrogen production

## Abstract

In the present work, an Ir/CeO_2_ catalyst was prepared by the deposition–precipitation method and tested in the decomposition of hydrazine hydrate to hydrogen, which is very important in the development of hydrogen storage materials for fuel cells. The catalyst was characterised using different techniques, i.e., X-ray photoelectron spectroscopy (XPS), transmission electron microscopy (TEM), scanning electron microscopy (SEM) equipped with X-ray detector (EDX) and inductively coupled plasma—mass spectroscopy (ICP-MS). The effect of reaction conditions on the activity and selectivity of the material was evaluated in this study, modifying parameters such as temperature, the mass of the catalyst, stirring speed and concentration of base in order to find the optimal conditions of reaction, which allow performing the test in a kinetically limited regime.

## 1. Introduction

One of the possible solutions to overcome the main challenges regarding the safety and economy of hydrogen as an energy carrier is the use of liquid-phase chemical hydrogen storage systems for on-board hydrogen production [1]. One of the most promising liquid-phase hydrogen storage material that has been proposed is hydrous hydrazine [2].

Anhydrous hydrazine is a well-known monopropellant for rockets due to its rapid decomposition, in particular on Ir/Al_2_O_3_ catalysts [3]. NH_2_NH_2_ has a high hydrogen content of 12.5 wt%, and it is an oily liquid at room temperature. Anhydrous hydrazine easily decomposes on a series of different materials from noble metals to metal carbides and nitrides, producing a large volume of gas and potentially hydrogen if the temperature reached is high enough, increasing, therefore, the risk of fire and explosion during the storage [4].

In order to avoid any explosive danger related to the storing of hydrazine, the use of the monohydrate form is preferred to the anhydrous counterpart. Hydrazine monohydrate (hydrous hydrazine), despite the addition of one mole of water for each one of hydrazine, maintains a hydrogen content of 7.9 wt%, above most of the requirement for hydrogen storage material, and it is more stable [5]. Moreover, it has the tendency to decompose to ammonia, reducing the explosion risk during storage. NH_2_NH_2_·H_2_O is liquid between −60 and 120 °C; this characteristic makes it a promising candidate for the replacement of gasoline in the already existent recharging networks for internal combustion engines [5].

Hydrous hydrazine can decompose following two main reaction pathways: (i) decomposition to hydrogen and nitrogen (Equation (1), complete decomposition) and decomposition to ammonia and nitrogen (Equation (2), partial decomposition) [6]. The complete decomposition toward hydrogen is the desired reaction pathway for application in fuel cells, leading to the highest hydrogen yield without the formation of undesired and harmful ammonia that can lead to catalyst deactivation and environmental pollution. Nonetheless, the production of ammonia is thermodynamically favourable. Therefore, the development of a catalyst is necessary to activate but also selectively decompose hydrous hydrazine to hydrogen and nitrogen with high yield, following the first reaction pathway and avoiding the formation of ammonia as indicated in the second reaction pathway.

(1) $${\mathrm{NH}}_{2}{\mathrm{NH}}_{2}\to N_{2}+2H_{2}\;∆H=-94.5{\;\mathrm{kJ}\;\mathrm{mol}}^{-1}$$

(2) $$3{\mathrm{NH}}_{2}{\mathrm{NH}}_{2}\to N_{2}+4{\mathrm{NH}}_{3}\;∆H=-157{\;\mathrm{kJ}\;\mathrm{mol}}^{-1}$$

Catalytic studies on the decomposition of hydrous hydrazine have been performed using mainly unsupported and supported metal nanoparticles as heterogeneous catalysts [7,8,9] in the presence of a base. Hydrazine is Brønsted base capable of accepting a proton from water [10].

(3) $${\mathrm{NH}}_{2}{\mathrm{NH}}_{2}+H_{2}O\to {\mathrm{NH}}_{2}{\mathrm{NH}}_{3}^{+}+{\mathrm{OH}}^{-}$$

(4) $${\mathrm{NH}}_{2}{\mathrm{NH}}_{2}\to {\mathrm{NH}}_{2}{\mathrm{NH}}^{-}+H^{+}$$

This interaction, Equation (3), with the reaction medium increases the presence of N_2_H_5_^+^ in the solution, which, after a first mechanist step of cleavage of the N–N, leads to a preferential formation of NH_3_, competing with the deprotonation of hydrazine (Equation (4)) [11,12]. For this reason, in literature, the use of a base as an additive to the reaction medium is often reported to increase the selectivity of the system to hydrogen generation. Moreover, the use of an alkaline solution increases the basicity of the surface of the catalyst leading to an inhibiting effect on the formation of basic ammonia [11].

Singh et al. reported the activity of a series of monometallic nanoparticles (Rh, Co, Ru, Ir, Cu, Ni, Fe, Pt and Pd) as catalysts for hydrazine decomposition. Among those, Cu, Ni, Fe and Pt were inactive for the decomposition while the other metals produced gases. However, while Co, Ir and Ru showed only 7% of selectivity toward hydrogen, Rh nanoparticles were able to achieve 43.8% of selectivity to the desired reaction at 25 °C [13].

Moreover, Jang et al. [14] have prepared Ir NPs supported on γ-Al_2_O_3_, prepared with repeated soak-dry processes obtaining raft-like and spherical shape nanoparticles increasing the metal content (from 5.8 to 16%). They demonstrated that hydrazine hydrate could decompose rapidly near ambient temperature on the 2% Ir honeycomb catalyst. Furthermore, a catalytic system was designed in order to recover the catalyst confirming the stability of the material.

Additionally, our group reported a DFT study regarding the decomposition of hydrazine over an Ir(111) surface, underlining the ability of the Ir catalyst to decompose this substrate. The investigated material showed a preference for the incomplete decomposition of hydrazine, forming mostly NH_3_ and N_2_. It was also pointed out that controlling the anti-bonding molecular orbital (π*) of the system can change the selectivity of the catalyst [15].

Furthermore, the influence of metal oxide support has been studied over the years. For example, Huang et al. deposited a small amount of CeO_2_ on Ni nanoparticles, which demonstrated the existence of a strong metal–support interaction between Ni and CeO_2_. CeO_2_ presented a ratio Ce^+3^/Ce^+4^ of 1.33, indicating a high number of vacancies in the oxide. The authors attributed the increased activity due to the presence of these vacancies that can perturb the electronic structure of Ni, therefore, modifying the interaction between nickel and hydrous hydrazine and finally increasing the selectivity to molecular hydrogen to 99%. In the same work, CeO_2_ was reported to have the best beneficial effects on the catalytic properties of Ni among other oxides, such as La_2_O_3_, MgO and ZrO_2_. All of the systems mentioned above are examples of unsupported nanoparticles catalysts. These systems have the advantage of better contact between particles and reagents, but their separation from the reaction mixture has a higher degree of difficulty; for this reason, supported nanoparticles are usually preferred to be utilised for chemical processes [16,17,18,19].

In the present study, we report the catalytic performance of Ir/CeO_2_, synthesised by the deposition-precipitation method, and the effect of reaction conditions on the activity and selectivity of the catalytic decomposition of hydrous hydrazine to hydrogen. Modifying parameters, such as temperature, the mass of the catalyst, stirring speed and concentration of base, the optimal conditions of reaction can be found, which allow performing the test in a kinetically limited regime.

## 2. Materials and Methods

### 2.1. Catalyst Preparation

**Figure 1:**
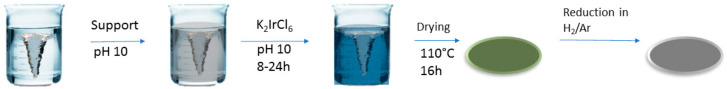
Schematic representation of the deposition—precipitation method with NaOH for Ir catalysts.

The synthesis of Ir/CeO_2_ catalysts was based on the deposition–precipitation method using NaOH. Firstly, in a 100 mL beaker, 50 mL of distilled water and the support were added and stirred. The dispersion of the support in water was then treated with a NaOH solution of 0.1 M dropwise until the desired pH reached the value of 10. When the pH of the dispersion was stable, the desired amount of the Ir precursor (K_2_IrCl_6_, Alfa Aesar, Kandel, Germany 99% purity) was added (nominal Ir loading 1%). The pH was adjusted continuously to 10 by adding NaOH aqueous solution dropwise, and it was kept constant at that value for 8 h, which was necessary for the full theoretical deposition of Ir on the surface of the support (CeO_2_, Sigma-Aldrich, Darmstadt, Germany) (Figure 1). The catalyst was then filtered, dried in an oven at 110 °C and finally reduced at 600 °C to obtain metallic nanoparticles. Different experiments were performed in order to select the optimised temperature of reduction. The maximum superficial Ir^0^ amount was reached at 600 °C (Figure S1). The heat treatment was performed in a horizontal furnace, equipped with a quartz tube, capable of reaching temperatures as high as 800 °C using a calcination boat in ceramic to contain the catalyst powder during the treatment. A reduction step was performed under a flow of 10% H_2_ in Ar, 80 mL min^−1^, with a temperature increase of 10 °C/min until a heat-treatment temperature of 600 °C was reached. After reaching the final heat-treatment temperature, the sample was kept at the desired temperature for 3 h.

### 2.2. Catalyst Characterisation

A Thermo Scientific K-α+ spectrometer (Waltham, MA, USA) was used to perform X-ray photoelectron spectroscopy (XPS) analyses. The spectrometer uses a monochromatic Al X-ray source operating at 72 W (6 mA × 12 kV). The signal was averaged on an oval-shaped area of approximately 600 × 400 microns. Data were recorded using pass energies of 150 eV for survey scans and 40 eV for high-resolution scans with a 1 and 0.1 eV step, respectively. Charges on the samples were neutralised using a combination of low energy electrons and argon ions (less than 1 eV) in order to have a C 1 s binding energy for adventitious carbon of 284.8 eV. The experimental spectra were fitted after subtraction of Shirley [20,21] or U2 Tougaard background using CasaXPS (v2.3.17 PR1.1) and Scofield sensitivity factors with an energy exponent of −0.6.

Particle size distributions and mean nanoparticle size was obtained performing Transmission Electron Microscopy using a JEOL JEM 2100 TEM (Akishima, Tokyo, Japan) operating at 200 keV. For examination, powder samples were dispersed in high purity ethanol and a drop of the suspension was deposited on a holey carbon film supported by a 300-mesh copper TEM grid and then left to evaporate. In order to obtain a reliable mean nanoparticle size of the desired metal nanoparticles and particle size distributions determination, at least 200 particles from different areas were measured.

The sample was analysed by scanning electron microscopy performed by a Tescan Maia3 field emission gun scanning electron microscope (FEG-SEM, Brno, Czech Republic) fitted with an Oxford Instrument XMAXN 80 energy dispersive X-ray detector (EDX). Images were acquired using the secondary electron and backscattered electron detectors. Samples were dispersed as a powder onto 300 mesh copper grids coated with holey carbon film.

The relative elemental composition can be assessed by EDX since the quantity of X-rays emitted at specific energy values is proportional to the amount of each specific atom.

ICP-MS analyses of solutions of the preparation of a catalyst after the filtration and separation of the solid catalyst was performed to quantify the amount of metal that was not immobilised/deposited during the preparation of the catalysts and, therefore, remained in the solution. The analysis was performed on an Agilent 7900 ICP-MS (Santa Clara, CA, USA) with full calibration for Ir (1, 0.1, 0.01, 0.001 mg L^−1^ and blank) prepared with certified standards from water. The analyses were performed on the collected filtrated solution after washing the solid with an exact amount of deionised water so that the total volume is known. Therefore, the amount of metal that remained in the solution can be calculated by multiplying the volume of the solution by the concentration found by the ICP-MS analyses.

### 2.3. Catalytic Tests

The typical catalytic tests were performed in a sealed batch reactor. The decomposition of hydrous hydrazine was followed either by the volume of gas produced and then collected or by the pressure changes inside a completely sealed reactor. Batch reactions were performed to obtain kinetics data in an accurate and reproducible way.

The set-up was composed of a single-neck sealed cap round bottom flask used as a reactor. The typical experimental protocol for the reaction was the following: the weighted mass of the desired catalyst was added in the reactor with 8 mL of a NaOH solution in water, which was varied from 0 to 1 M, and a magnetic stirrer bar. The reactor was placed in an oil bath on a magnetic stirring hot plate, which was set to reach a chosen temperature. The temperature of the reaction varied between 30 and 70 °C. While the reactor was warming, the valve on the lid was kept closed to avoid evaporation of the aqueous solution. When the reaction temperature was reached and was stable, the magnetic stirrer was quickly stopped to allow the addition of 0.3 mL of 3.3 M hydrous hydrazine solution. Then, the system was purged with N_2_ gas in order to eliminate O_2_ and, therefore, maintain the gas mixture outside the explosion limits of H_2_. The valve on the lid of the reactor was then connected via flexible tubes to a washing bottle filled with 1 M HCl solution and, after, to a burette filled with water where the gasses could be collected.

Once the lid was connected to the gas collection system, the valve on the lid was open, and the stirring was initiated again in order to start the reaction. The acid washing bottle was used to trap possible ammonia generated during the decomposition and evaporated so that the volume read from the displacement was related only to H_2_ and N_2_. The volume of gas produced was quantified using a burette filled with water and rotated upside-down in a beaker filled with water. When the gas is collected in the burette, it displaces the water; therefore, from the variation in the position of the meniscus in the burette, the total volume of gas produced can be quantified. From the volume of gas produced using the ideal gas law, as shown in Equation (5), it is possible to calculate the moles of H_2_ and N_2_.

(5) pV=nRT

Van der Waals corrections were tried too, but these corrections did not vary the number of moles calculated at the working condition of the setup significantly. The reaction was then performed more than once in order to guarantee its reproducibility. At the end of the reaction, the remaining hydrous hydrazine in the solution was quantified by the colourimetric method.

The yield of the reaction was then calculated by a formula derived from the two stoichiometric reactions, Equations (6)–(8) [22].

(6) $$N_{2}H_{4}\to N_{2}+2H_{2}$$

(7) $$3N_{2}H_{4}\to N_{2}+4{\mathrm{NH}}_{3}$$

(8) $$3N_{2}H_{4}\to \left(1+2X\right)N_{2}+6{\mathrm{XH}}_{2}+\left(4-4X\right){\mathrm{NH}}_{3}$$

where X is the selectivity of the reaction to hydrogen, with a value of one for complete decomposition to hydrogen. Since ammonia, if produced, is trapped using the washing bottle filled with acid and the moles N_2_ and H_2_ have to be considered as moles of total gas, it is not possible to distinguish the moles of the two gasses from the volume collected (Equation (10)); thus, the moles of gas are quantified and, therefore, ammonia is then removed from the equation (Equation (9)).

(9) $$3N_{2}H_{4}\to \left(1+2X\right)N_{2}+6{\mathrm{XH}}_{2}$$

(10) $$3N_{2}H_{4}\to \left(1+8X\right)\mathrm{gas}$$

Later, the moles of gas produced, n(H_2_ + N_2_), from the moles of hydrazine consumed, n(N_2_H_4_), is calculated.

(11) $$\frac{n(N_{2}H_{4})}{3}=\frac{n\left(H_{2}+N_{2}\right)}{\left(1+8X\right)}$$

(12) $$\left(1+8X\right)=3\frac{n\left(H_{2}+N_{2}\right)}{n(N_{2}H_{4})}$$

When the decomposition is completed, the n(H_2_ + N_2_)/n(N_2_H_4_) parameter reaches a value of 1/3, in the case of complete decomposition to ammonia (X = 0), and 3, in the case of complete decomposition to hydrogen (X = 1), as can be calculated from Equation (10). At the same time, the selectivity to hydrogen, X, at full conversion is equivalent to the value of the yield to hydrogen, Y [23].

(13) $$Y=\frac{3\frac{n\left(H_{2}+N_{2}\right)}{n(N_{2}H_{4})}-1}{8}$$

For the analysis of the gas produced by the decomposition reaction, the reactor was connected to a gas sealed bag instead of the gas burette so that the gas could be fed later to the portable mass spectrometer in order to perform gas quantification via mass spectroscopy.

Reusability tests were performed following the same experimental procedure of the batch reaction quantified by the volumetric method. From the second reaction, fresh hydrous hydrazine solution was added in order to restart the experimental test, while the catalyst was kept in the reaction solution between the reaction cycles.

## 3. Results

ICP-MS was used to quantify the amount of Ir deposited on the CeO_2_ support during the deposition step of the preparation method. The result indicates that 69.9 wt%. of Ir was deposited on the surface. The metal loading calculated by ICP-MS is, therefore, used to calculate the substrate/metal molar ratio and TOF values for the catalytic tests.

### 3.1. Catalytic Decomposition of Hydrous Hydrazine

In order to provide accurate and reproducible sets of kinetic data, the hydrous hydrazine decomposition tests were performed in a batch reactor under optimised reaction conditions.

To ensure that the tests were reproducible, the reactions were performed in the same reaction condition twice or three times, as exemplified in Figure S2, where the value of n(H_2_ + N_2_)/n(N_2_H_4_) is plotted against the reaction time.

As it can be observed, the initial values of n(H_2_ + N_2_)/n(N_2_H_4_) are the most affected by the experimental error of measurements. This is due to both a larger effect of the experimental error of the blank reaction, which tends to be more stable on longer reaction times, and a general larger relative error that decreases when the value of the measurement increases, with the same absolute error remaining on the measurement over the duration of the reaction.

An accurate evaluation that the catalytic activity has been performed under kinetic conditions was carried out.

### 3.2. Effect of Stirring Rate

**Figure 2:**
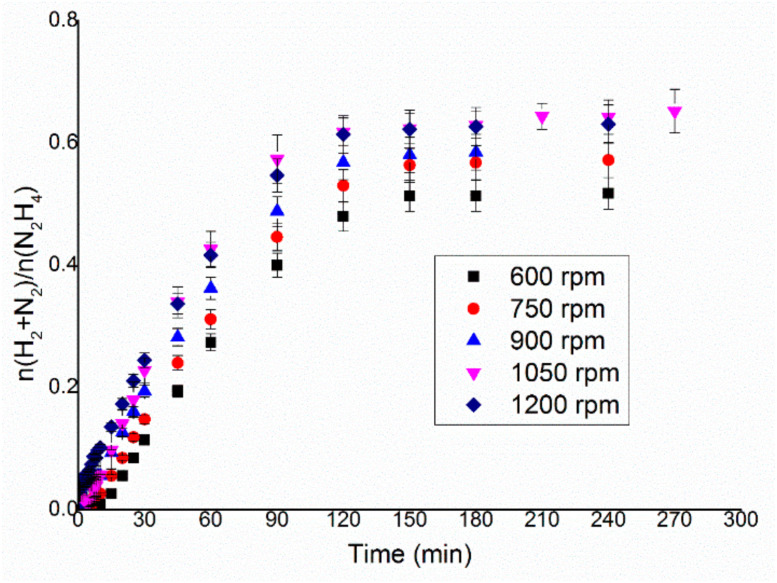
n(H_2_ + N_2_)/n(N_2_H_4_) versus time for hydrous hydrazine decomposition over Ir/CeO_2_ using 0.3 mL of 3.3 M hydrazine monohydrate in 8 mL 0.5 M of NaOH solution, at 50 °C and 152.4 mg of catalyst, varying the stirring rate from 600 to 1200 rpm.

**Figure 3:**
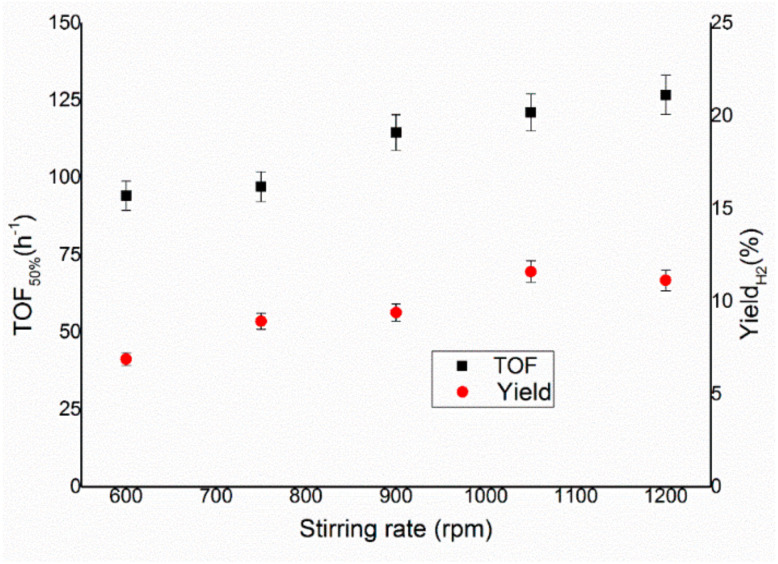
Turnover frequency, left y–axis, and yield toward hydrogen, right y–axis, of the reaction over Ir/CeO_2_ at different stirring rates.

External mass limitations are related to the diffusion of the reactants through the interphase layer between liquid and solid. This is influenced by three main factors: the surface of the interphase, the concentration of the species on the two phases and the thickness of the interphase itself. The surface is strictly linked with the amount of catalyst used and the dimension of the particles, which can be considered constant for the same catalyst and amount. The initial concentration of the hydrous hydrazine in the solution was kept constant for reasons related to the experimental setup used. The stirring rate was consequently varied in the range between 600 and 1200 rpm, as shown in Figure 2, whereas the temperature and the substrate/metal molar ratio were kept at 50 °C and 250:1 substrate/metal molar ratio and 0.3 mL of 3.3 M hydrous hydrazine solution in 8 mL of 0.5 M NaOH were used. From the comparison of the TOF_50%_ using different stirring rates (Figure 3), a plateau was reached for values above 900 rpm, confirming the achievement of kinetic regimes above this stirring rate.

The gas evolution as a function of time, displayed in Figure 2, showed how in this case the variation of the stirring rate did not affect only the rate of the reaction, but at a lower stirring rate, the final yield toward the molecular hydrogen of the system was lower. For both parameters, such as reaction rate and selectivity, 1050 and 1200 rpm were equally feasible stirring rates to be used in the hydrous hydrazine decomposition catalytic tests, as it is shown in Figure 3. To decide which of the two rates to use in the succeeding tests, the stability of the rotation of the stirrer during the reaction was considered. Higher stirring speed decreases the stability of the magnetic stirrer on the rotation axis, which can lead to droplets of the solution on the walls of the reactor and general instability of the stirrer bar. For these reasons, 1050 rpm was chosen as the optimal stirring rate for the following catalytic tests.

### 3.3. Effect of Mass of Catalyst

**Figure 4:**
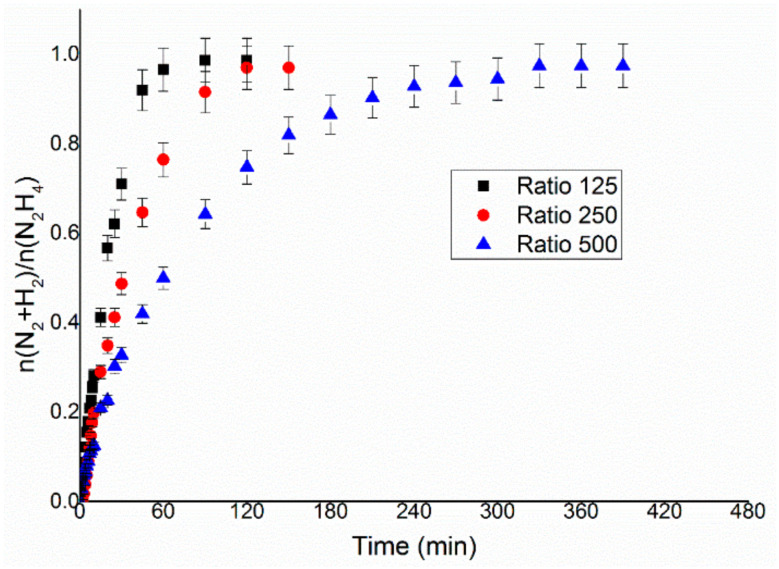
n(H_2_ + N_2_)/n(N_2_H_4_) versus time for hydrous hydrazine decomposition over Ir/CeO_2_ using 0.3 mL of 3.3 M hydrazine monohydrate in 8 mL 0.5 M of NaOH solution, at 50 °C and a stirring rate of 1050 rpm, with different amounts of catalyst 38.1, 76.2 and 152.4 mg for the ratios 500:1, 250:1 and 125:1, respectively.

The preliminary tests on the influence of the mass of the catalyst used a temperature of reaction of 50 °C, 0.3 mL of hydrous hydrazine 3.3 M in 8 mL of 0.5 M NaOH solution. The reaction mixture was left to react until the production of gases was stopped for at least 30 min. The stirring rate used for these tests was set to a value of 1050 rpm following the study reported in the previous section. These catalytic tests were performed varying the amount of catalyst used from 38.1 to 152.4 mg, which correspond to a substrate/metal molar ratio of 500:1 to 125:1 (Figure 4). This range of values was chosen for two reasons; firstly, molar ratios lower than 125:1 requires high amounts of catalyst, which would have precluded the possibility of higher the number of catalytic tests on these catalysts; secondly, for molar ratios lower than 500:1, following the trend demonstrated by the catalytic tests performed, would have required a longer reaction time to record good readings of the data in the final stages of the reaction. The reaction profiles of these catalytic tests, displayed in Figure 4, demonstrate that the amount of catalyst used did not have any sensible influence on the final yield of the reaction at the specific studied range. However, in order to identify a possible influence of mass transfer effect on the value of TOF_50%_, it was calculated in order to weigh the activity of the reaction with the amount of active metal used in the reaction. These TOF_50%_ have been calculated to be 297.6, 358.3 and 366.0 h^−1^ for the ratios 125:1, 250:1 and 500:1, respectively, whereas the final yields are 24.5%, 23.9% and 24.1%, which can be considered constant within the experimental error of the measurement. These data showed that, as expected, the final yield of the reaction is independent of the mass of catalysts used in the reaction, but the activity increases between the ratios 125:1 and 250:1, while the differences in TOF between 250:1 and 500:1 stay within the error of the analyses. The observed catalytic behaviour indicated the higher amount of catalysts (ratio 125:1) tend to limit the diffusion of the reagent in the solution, whereas the kinetic regime was achieved above the 125:1 molar ratio.

The 250:1 substrate/metal molar ratio, corresponding to 76.2 mg in the case of 1 wt% Ir catalysts, was chosen as the optimal value of the following catalytic tests, as a reasonable compromise between the time of reaction and the amount of catalyst used, allowing to perform at least two catalytic reactions per day per experimental setup and more than 10 reactions per batch of catalyst synthesised.

### 3.4. Effect of NaOH Concentration

**Figure 5:**
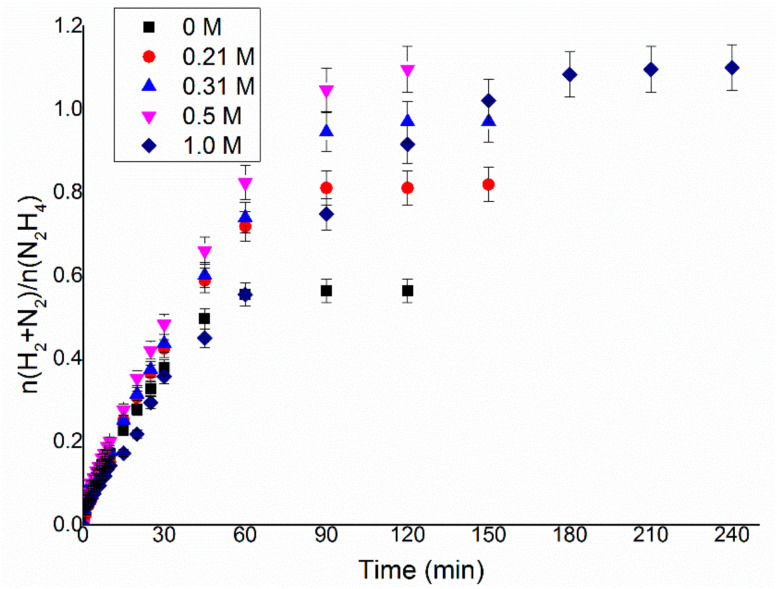
n(H_2_ + N_2_)/n(N_2_H_4_) versus time for hydrous hydrazine decomposition over Ir/CeO_2_ using 0.3 mL of 3.3 M hydrazine monohydrate in 8 mL of NaOH solution with a concentration between 0 and 1.0 M, at 50 °C, stirring rate of 1050 rpm and a 250:1 substrate to meta molar ratio.

**Figure 6:**
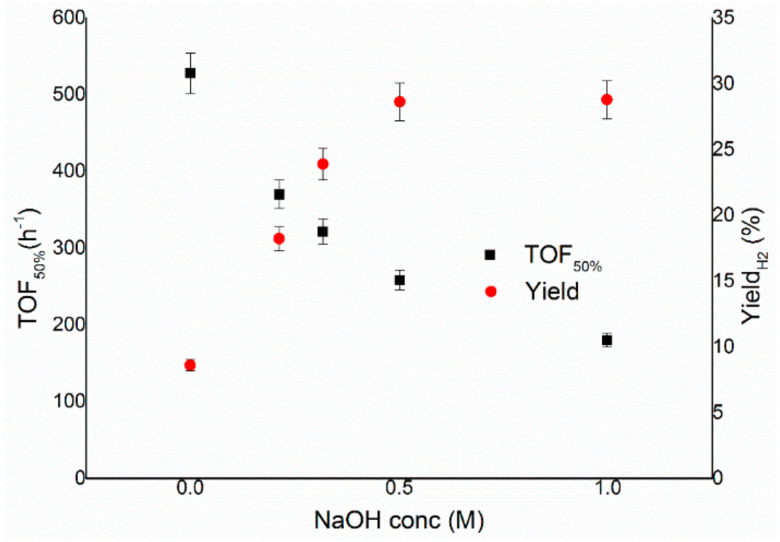
Turnover frequency, left y–axis, and yield toward hydrogen, right y–axis, of the reaction over Ir/CeO_2_ as a function of the concentrations of NaOH.

As the final step of the optimisation process, the influence of the concentration of sodium hydroxide on the reaction solution was studied. Previous works in the literature have displayed that the presence of NaOH in the decomposition of hydrous hydrazine in aqueous solution has a beneficial effect on the selectivity of the reaction, and in some cases also on the activity of catalysts [11,24,25]. This effect is most probably related to the formation, in neutral solutions, of the protonated form of hydrazine (N_2_H_5_^+^). The presence in N_2_H_5_^+^ of a third hydrogen atom bonded to a nitrogen probably leads to the direct formation of ammonia on the surface of the metal, which is more stable than other species derived from N_2_H_4_ and, therefore, decreases the formation of molecular hydrogen. Hence, the effect of the concentration of NaOH in solution was studied, varying its value between 0 and 1 M, and results are presented in Figure 5, using the previously optimised values of a substrate/metal molar ratio of 250:1 and a stirring rate of 1050 rpm at 50 °C and using Ir/CeO_2_ as the reference catalyst. Figure 6 displays the increase in final yield with the concentration of sodium hydroxide until a plateau is reached around a concentration value of 0.5 M, whereas the activity of these catalysts decreases with the decrease of the concentration of sodium hydroxide.

The observed catalytic behaviour can be due to different explanations, such as (I) the lower rate of adsorption of the reagents and/or (II) the slower individual elemental steps. With the aim of achieving the highest selectivity possible from the catalytic system using the lowest possible amount of additive, to increase the economy of the system itself, it was decided to perform the consequent tests with a NaOH concentration of 0.5 M since this was the lowest value on the plateau of the yield versus concentration, as shown in Figure 6.

### 3.5. Effect of Reaction Temperature

**Figure 7:**
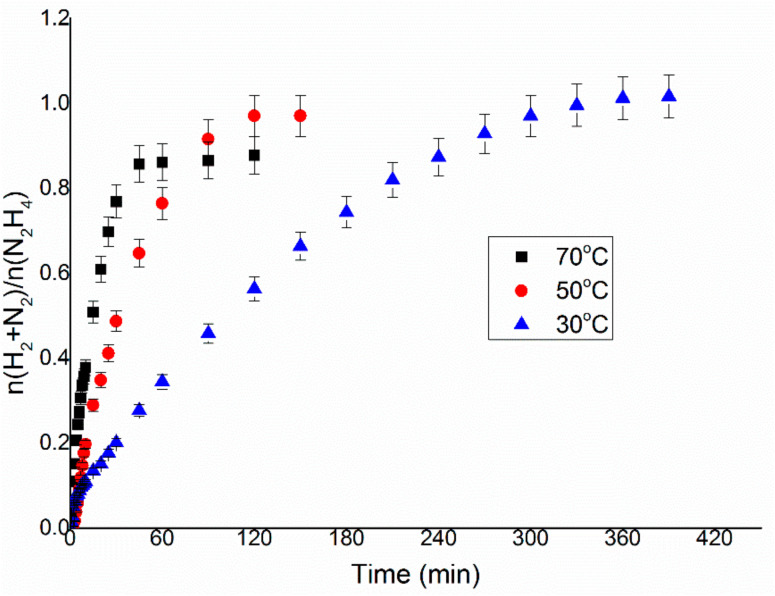
n(H_2_ + N_2_)/n(N_2_H_4_) versus time for hydrous hydrazine decomposition over Ir/CeO_2_ using 0.3 mL of 3.3 M hydrazine monohydrate in 8 mL of 0.5 M NaOH solution at a stirring rate of 1050 rpm and a 250:1 substrate to metal molar ratio at temperatures of 30, 50 and 70 °C.

The last parameter to optimise for the liquid phase catalytic decomposition of hydrous hydrazine was the temperature of the reaction. In the literature, it is reported that for many catalysts, the increase of the temperature of the reaction mixture led to a decrease in the final yield of hydrogen of the system, whereas the activity increased [26,27]. To confirm this trend, the reaction was performed at 30, 50 and 70 °C with the experimental conditions optimised in the previous reported catalytic tests, as shown in Figure 7.

**Figure 8:**
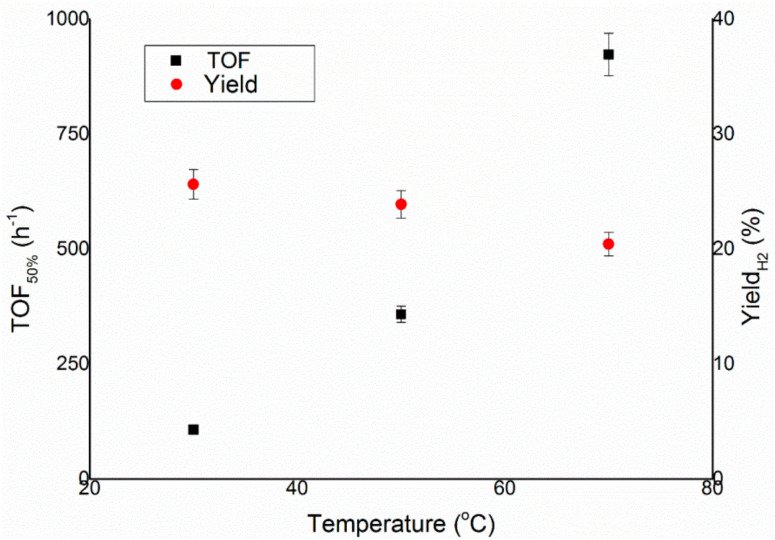
Turnover frequency, left y–axis, and yield toward hydrogen, right y–axis, of the reaction over Ir/CeO_2_ as a function of temperature.

Figure 8 displays that the tendency of the decrease of selectivity is respected also in the case of Ir/CeO_2_, where the final yield decreased from 25.6% at 30 °C to 20.4% at 70 °C. On the other hand, the TOF_50%_ of this reaction went from 107.3 to 923.0 h^−1^ for 30 and 70 °C. The temperature chosen for the succeeding tests was 50 °C, which showed values of intermediate activity and selectivity, permitting to also test catalysts with lower activity without sacrificing much in terms of selectivity.

### 3.6. Reusability Studies

**Figure 9:**
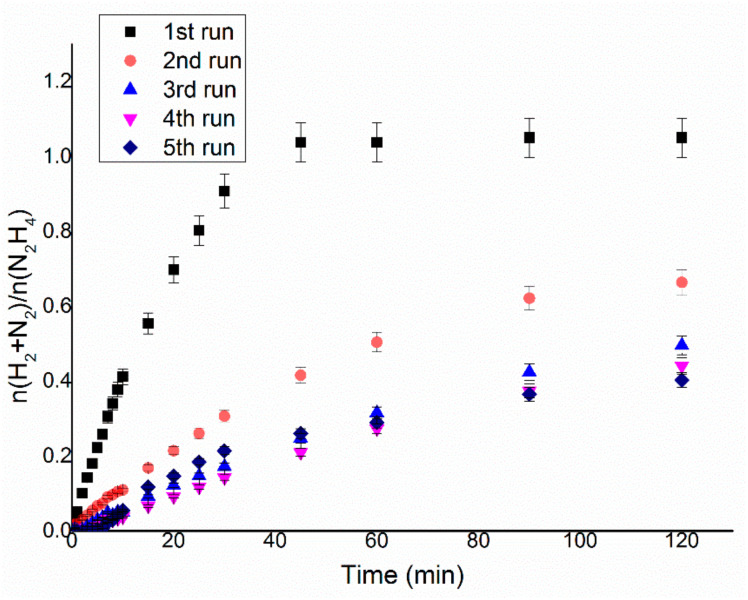
n(H_2_ + N_2_)/n(N_2_H_4_) versus time for hydrous hydrazine decomposition over Ir/CeO_2_ using 8 mL of 0.5 M NaOH solution at 50 °C, a stirring rate of 1050 rpm and a 250:1 substrate to metal molar ratio. A total of 0.3 mL of 3.3 M hydrazine monohydrate was added before each reaction.

In order to assess the stability of the catalytic material under the experimental reaction conditions, a preliminary reusability test was performed on Ir/CeO_2_. Reusability tests were conducted by adding a fresh hydrous hydrazine solution of known concentration when the gases stopped evolving from the reaction mixture, which is considered a signal of complete decomposition of hydrous hydrazine (100% conversion). The same catalyst was used in up to five reaction cycles, as can be seen in Figure 9.

**Figure 10:**
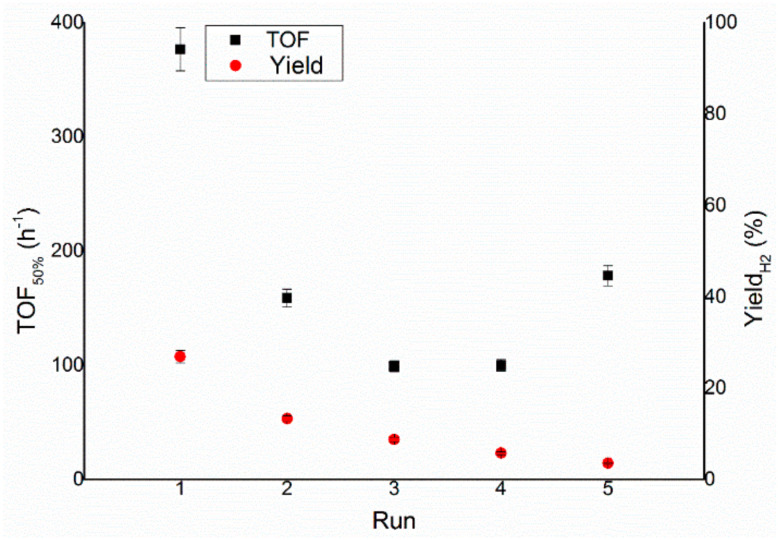
Turnover frequency, left y–axis, and yield toward hydrogen, right y–axis, of the reaction over Ir/CeO_2_ during the reusability tests.

The final yield of the reaction toward molecular hydrogen decreases after each run, whereas the activity measured by the TOF_50%_ reaches a minimum in the third run, as shown in Figure 10. Firstly, to be noticed is that the reaction restarts when a new aqueous solution of hydrous hydrazine is added in the sealed reactor, indicating a preliminary proof in this way that at the end of the gas evolution is related to a complete decomposition of the reagent; this assumption was later verified during the project by colourimetric quantification of hydrazine in the reaction mixture. Secondly, the variations in terms of activity and the decrease in final yield during the subsequent reactions can indicate a possible change of the catalytic species presented on the surface of the support. The aqueous catalytic decomposition of hydrous hydrazine can also take multiple pathways for the production of the same products, so the poisoning of specific catalytic sites [28] or the reconstruction [29] of the surface can have various specific pathways with respect to the others. The increase of the TOF between the third, fourth and fifth runs strongly suggests a mechanism of modification of the surface since the creation of the type of catalytic active species on the surface of the nanoparticles would agree with a change of reaction pathway for the production of hydrogen, for example, the one exhibited by the Ir/CeO_2_ catalyst in this specific reaction test. This modification of the nanoparticles would agree with the comparison among preparation methods that produce small mean particle size and larger mean particle size, in which the latter shows higher activity but lower selectivity than the material with a smaller mean particle size.

**Figure 11:**
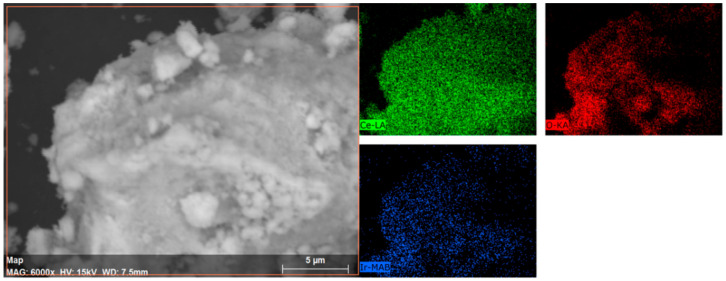
SEM-EDX mapping of a portion of the Ir/CeO_2_ sample, which displays a homogeneous distribution of Ce, O and Ir across the material as expected.

In order to better understand the catalytic materials tested, a series of different characterisation techniques and measurements were performed to determine Ir mean particle size and particle size distribution, superficial oxidation state and surface coverage. Figure 11 shows a representative image of Ir/CeO_2_ catalyst morphology on the 10 µm scale. From the EDX, the average total value of Ir loading onto the support is 0.70 wt% ± 0.1 with a good dispersion over the sample, with no empty zone of Ir nanoparticles in the Ir map. The 0.70 wt% is lower than the calculated 1.0 wt% by the amount of precursor used during the preparation of the catalyst, but it agrees with the ICP-MS analyses previously reported (0.69 wt%). This lower loading is probably related to the deposition–precipitation method employed for the synthesis, which can be greatly influenced by pH, temperature and time of deposition.

Ir/CeO_2_ was analysed both before (fresh) and after (used) the reaction. In Figure S3, representative images of the fresh and used catalysts are displayed. As can be seen, the CeO_2_ is present as crystals in the range of 10–50 nm. On the other hand, only a few nanoparticles of Ir can be distinguished from the background due to low contrast with the support, e.g., only nanoparticles on a thin layer of oxide or on the edge of its particles, and also because their dimensions are small.

The fresh catalyst, Ir/CeO_2_, has a mean particle size of 0.9 ± 0.2 nm, whereas the same catalyst, after being used, has a mean particle size of 1.2 ± 0.4 nm. The difference between the mean particle size of fresh and used catalysts is in the range of the error of the analyses.

**Figure 12:**
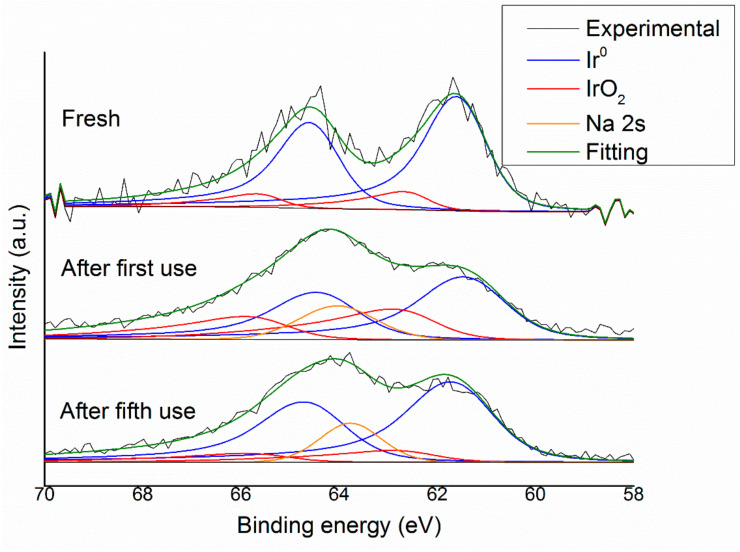
XPS experimental spectra and fitting of Ir 4f for Ir/CeO_2_ fresh and after one and five uses with the deconvolution of the peaks.

XPS analyses have been performed on Ir/CeO_2_ before and after the reaction and also at the end of the reusability study, i.e., after a cycle of 10 reactions in a row (Figure 12).

All the iridium species in the Ir 4f region display doublet spins due to the spin-orbit splitting that leads to the creation of the Ir 4f_7/2_, in the range 60.8–62.8 eV, and Ir 4f_5/2_, in the range of 63.8–65.8 eV. Moreover, the area of the two peaks of the doublet is related by a specific ratio that in the case of the 4f orbital is 3:4 for 5/2:7/2 peaks. In particular, using the Ir 4f_7/2_ as the reference, in the literature, metallic Ir is reported to have a binding energy of 60.9–61.2 eV, whereas IrO_2_ has a binding energy of 61.9–62.5 eV and IrCl_3_ has one of 62.2–62.6 eV [30].

For these XPS spectra, the unfitted data showed a peak for the Ir 4f_7/2_ at 61.6–61.8 eV, which can mislead to the assignment of this as IrO_2_, but that would be in contrast with the TPR data discussed earlier that confirm the high degree of reduction of the iridium precursor. Therefore, they were fitted with two different peaks, and the range of binding energies used were enlarged at higher values than the ones reported in the literature in order to get a more precise fitting of the signal. The best fitting gives two peaks, one in the range 61.5–61.8 eV and the other 62.5–62.9 eV, which have been assumed to be Ir^0^ and Ir^+4^ and are higher than the literature values. Consequently, the peaks at 61.5–61.8 eV, which undergo an up shift, have been assigned to metallic iridium nanoparticles with partial positive charges due to the small dimensions and interaction with the support, and the peaks at 62.5–62.9 eV have been assigned to IrO_x_ species.

The comparison between a fresh and used catalyst, as shown in Figure 12, is less accurate due to the presence of traces of Na on the samples that are derived from the NaOH used in the catalytic reaction. The Na 2S orbital has a broad single peak at 63.0–64.0 eV; this overlaps with the Ir 4f region, and therefore, a precise quantification of the different Ir species is not possible, but the presence of peaks at 61.6–61.4 eV can also be found on the used samples. This small down-shift for the used samples may indicate that aggregation may have occurred and, therefore, an increase in the mean particle size. Taking into account the aforementioned observations, no final and ultimate conclusion can be derived due to the overlapping of the elements.

## 4. Conclusions

In the present work, an Ir/CeO_2_ catalyst prepared by the deposition–precipitation method was presented and tested in the decomposition of hydrazine hydrate to hydrogen, which is very important in the development of hydrogen storage material for fuel cells. The effect of reaction conditions on the activity and selectivity of the material was evaluated in this study, modifying parameters, such as temperature, the mass of the catalyst, stirring speed and concentration of base, in order to find the optimal conditions of reaction, which allow performing the test in a kinetically limited regime. The stirring rate was consequently varied in the range 600–1200 rpm. This parameter can influence both the reaction rate and selectivity of the system. In fact, both TOF and yield increase, increasing the stirring rate, reaching a plateau at 1050 rpm. These catalytic tests were performed varying the substrate/metal molar ratio from 500:1 to 125:1. In this case, the amount of catalyst used did not have any sensible influence on the final yield of the reaction, but the activity increases between the ratios 125:1 and 250:1. The 250:1 substrate/metal molar ratio was chosen as the optimal value as a reasonable compromise between the time of reaction and the amount of catalyst used. The effect of the concentration of NaOH in the solution was studied, varying its value between 0 and 1 M. An increase in final yield with the concentration of sodium hydroxide was observed until a plateau was reached around a concentration value of 0.5 M, whereas the activity of these catalysts decreased with the decrease of the concentration of sodium hydroxide. In order to achieve the highest selectivity possible from the catalytic system using the lowest possible amount of additive, a NaOH concentration of 0.5 M was selected as the optimal value. To conclude, the effect of temperature was also evaluated, performing tests at 30, 50 and 70 °C. A tendency of a decrease in selectivity increasing the T was observed for the catalysts, but the reaction rate increased with the temperature. For this reason, the temperature chosen was 50 °C, which showed values of intermediate activity and selectivity. Finally, a complete overview on the kinetic study was proposed in this paper in order to understand the perfect catalytic condition to apply in this system for hydrazine decomposition.

## Data Availability

The data presented in this study are available on request from the corresponding author.

## Supplementary Materials

The following are available online at https://www.mdpi.com/article/10.3390/nano11051340/s1, Figure S1: XPS experimental spectra and fitting of Ir 4f for Ir/CeO_2_ fresh at different reduction temperatures with the deconvolution of the peaks, Figure S2: n(H_2_ + N_2_)/n(N_2_H_4_) versus time for reaction of 0.3 mL, 3.3 M of hydrazine monohydrate in 8 mL 0.5 M of NaOH solution using 152.4 mg of Ir/CeO_2_ at 50 °C and 1050 rpm of stirring rate., Figure S3: TEM images of Ir/CeO_2_ fresh, a-d, and used, e-h. Ir nanoparticles can be seen as dark spots in the higher magnification images, while grey larger particles are the CeO_2_ support.
