# Supplementary material for: Hydrous Hydrazine Decomposition for Hydrogen Production Using of Ir/CeO2: Effect of Reaction Parameters on the Activity
Source: Nanomaterials (Basel). 2021 May 19;11(5):1340. doi: 10.3390/nano11051340 (PMC8161091; doi:10.3390/nano11051340)
Supplement: Supplementary file 1 [file nanomaterials-11-01340-s001.zip › nanomaterials-1214028-supplementary.pdf]

Supporting Materials

Hydrous hydrazine decomposition for hydrogen production using of Ir/CeO_2: effect of reaction parameters on the activity

Davide Motta [1], Ilaria Barlocco [2], Silvio Bellomi [2], Alberto Villa [2,*] and Nikolaos Dimitratos [3,*]

[1] Cardiff Catalysis Institute, School of Chemistry, Cardiff University, Main Building, Park Place, Cardiff CF10 3AT, United Kingdom; davide.motta90@gmail.com

[2] Dipartimento di Chimica, Università degli Studi di Milano, via Golgi 19, I-20133Milano, Italy; ilaria.barlocco@unimi.it (I.B.), silvio.bellomi@studenti.unimi.it (S.B.)

[3] Dipartimento di Chimica Industriale e dei Materiali, ALMA MATER STUDIORUM Università di Bologna, Viale Risorgimento 4, 40136 Bologna, Italy

* Correspondence: nikolaos.dimitratos@unibo.it (N.D.); alberto.villa@unimi.it (A.V.)

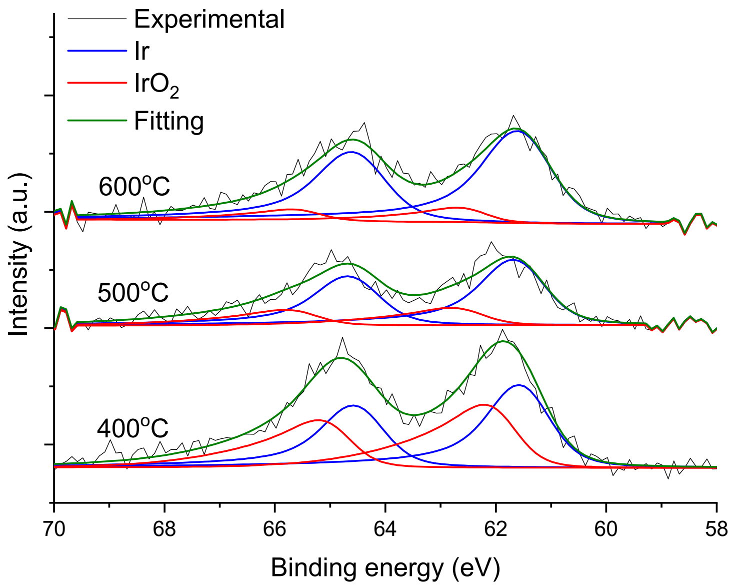

Figure S1. XPS experimental spectra and fitting of Ir 4f for Ir/CeO_2 fresh at different reduction temperatures with the deconvolution of the peaks.

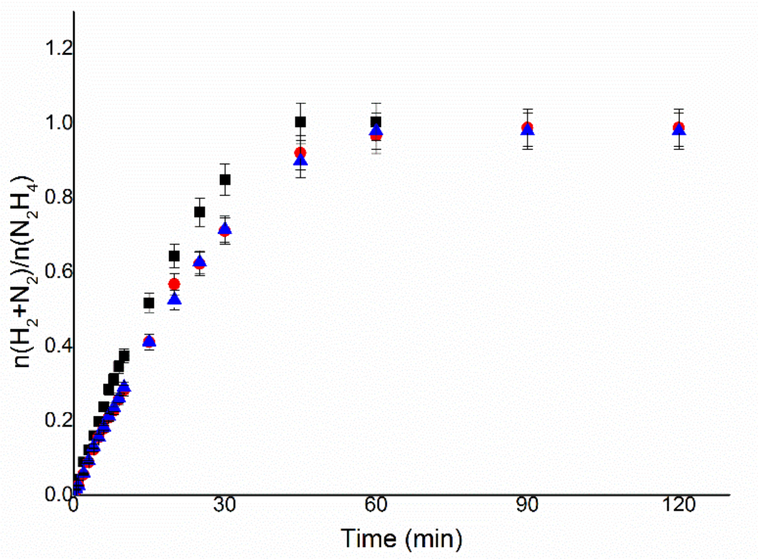

Figure S2. $n(H_2 + N_2)/n(N_2H_4)$ versus time for reaction of 0.3 mL, 3.3 M of hydrazine monohydrate in 8 mL 0.5 M of NaOH solution using 152.4 mg of Ir/CeO_2 at 50°C and 1050 rpm of stirring rate.

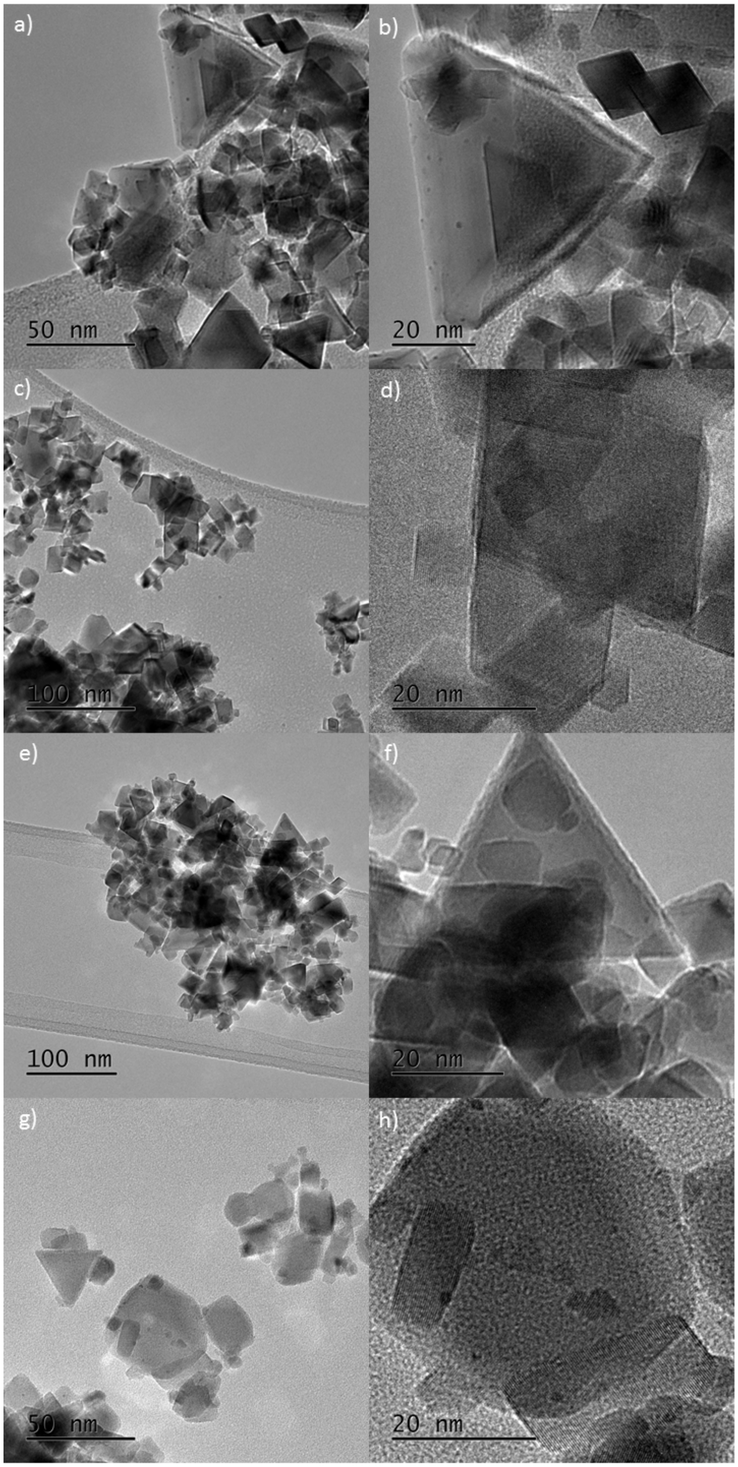

Figure S3. TEM images of Ir/CeO_2 fresh, a-d, and used, e-h. Ir nanoparticles can be seen as dark spots in the higher magnification images, while grey larger particles are the CeO_2 support.
